# On the potential roles of ticks and migrating birds in the ecology of West Nile virus

**DOI:** 10.3402/iee.v4.20943

**Published:** 2014-01-15

**Authors:** Karl Hagman, Christos Barboutis, Christian Ehrenborg, Thord Fransson, Thomas G. T. Jaenson, Per-Eric Lindgren, Åke Lundkvist, Fredrik Nyström, Jonas Waldenström, Erik Salaneck

**Affiliations:** 1 Section for Infectious Diseases, Department of Medical Science, Uppsala University, Uppsala, Sweden; 2 Hellenic Ornithological Society and Natural History Museum of Crete, Crete, Greece; 3 Swedish Museum of Natural History, Stockholm, Sweden; 4 Department of Systematic Biology, Medical Entomology Unit, Uppsala University, Uppsala, Sweden; 5 Department of Clinical and Experimental Medicine, Microbiology, Linköping University, Linköping, Sweden; 6 Department of Medical Microbiology and Biochemistry, Uppsala University, Uppsala, Sweden; 7 Swedish Institute for Communicable Diseases and Department of Microbiology, Tumor and Cell Biology, Karolinska Institutet, Solna, Sweden; 8 Center for Ecology and Evolution, Microbial Model Systems, Linneaus University, Kalmar, Sweden

**Keywords:** *West Nile virus*, *emerging infectious diseases*, *migratory birds*, *zoonoses*, *ticks*, Hyalomma marginatum *s.l*., Hyalomma rufipes

## Abstract

**Background:**

Mosquitoes are the primary vectors of West Nile virus (WNV). Ticks have, however, been suggested to be potential reservoirs of WNV. In order to investigate their role in the spread of the virus, ticks, which had been collected from birds migrating northwards from Africa to Europe, were analyzed for the potential presence of WNV-RNA.

**Methods:**

On the Mediterranean islands Capri and Antikythira a total of 14,824 birds were captured and investigated from which 747 ticks were collected.

**Results and conclusion:**

Most of the identified ticks (93%) were nymphs and larvae of Hyalomma marginatum sensu lato, most of which were or appear to be Hyalomma rufipes. Of these ticks 729 were individually screened for WNV-RNA. None of the ticks was found to be WNV positive. Thus, there was no evidence that Hyalomma marginatum s.l. ticks play a role in the spread of WNV from Africa to Europe.

West Nile virus (WNV) belongs to the genus *Flavivirus* of the family Flaviviridae (1). It is a mosquito-borne arbovirus with birds as the primary vertebrate host (2). Humans and other mammals are regarded as dead-end hosts. Infection in humans can lead to clinical disease, sometimes with central nervous system complications and high mortality rates, especially in older age groups (3).

WNV infection is considered to be an emerging infectious disease, and there have been numerous epidemics during the last 15 years (4). It is endemic in parts of Africa and epidemic in southern Europe (3). WNV was introduced to the Western Hemisphere as late as 1999 (5). Since then, it has spread over most of North America and has to date (1999–2011) caused more than 1,200 deaths in the United States alone (6). Migratory birds are regarded as the primary means for the virus to spread across the world (7).

Although mosquitoes are the primary vectors, the virus has been isolated repeatedly from both ixodid and argasid ticks, and ticks have been proposed as reservoirs for the virus during bird-associated transfer of the virus between geographical regions (2, 8, 9). In order to predict and control a potential further geographical spread of this virus, knowledge about its ecology is of outmost importance. To date, not enough data are available to assess the role of ticks in the maintenance and spread of the virus (10). This study aimed to investigate if ticks, which had infested migratory birds in Africa, may be infected with WNV when they arrive on their avian hosts in southern Europe. Thus, at two stopover sites, we net-captured birds that had just crossed the Mediterranean Sea, on their way from their wintering grounds in WNV-endemic Africa to their breeding grounds in Europe. A total of 729 ticks were collected from the birds and individually screened with polymerase chain reaction (PCR) for the presence of WNV RNA.

## Materials and methods

### Collection of ticks from birds

The birds were captured in mist nets at Capri bird observatory in Italy and at Antikythira bird observatory in Greece in two periods: 2 April–18 May 2009 and 11 March–19 May 2010. Each captured bird was identified to species, and its ears, throat, nape, and abdomen were checked for ticks (11, 12). All ticks observed were removed with forceps, photographed, individually submerged in Eppendorf tubes filled with RNA-later buffer (Qiagen, Hilden, Germany), frozen at -20°C, and stored in RNA-later for 6 months prior to RNA extraction.

### RNA extraction and cDNA synthesis

Ticks were homogenized using a Qiagen TissueLyzer (Qiagen) in tubes containing Buffer RLT (Qiagen) with 1% β-mercaptoethanol and a 5 mm steel bead for 2 min at 25 Hz. Each series of RNA extraction also included one NTC and one positive control spiked with Encepur^®^ tick borne encephalitis virus (TBEV) vaccine (Novartis Vaccines, Basel, Switzerland) and B31 *Borrelia* spirochetes. After homogenization, RNA extraction was performed in a Qiagen M48 BioRobot using the MagAttract^®^ RNA Tissue Mini M48 kit. The extracted RNA was stored in a -70°C freezer and later used for the WNV screening. Some of the extracted RNA was used for immediate cDNA synthesis. For this, we used a CAS-1200™ Precision Liquid Handling Robot (Corbett Research, Cambridgeshire, UK) to convert RNA into cDNA with the Illustra™ Ready-to-GO RT-PCR beads kit (GE Healthcare, Buckinghamshire, UK). Random hexamer primers pd(N)6 were used to ensure that total RNA was converted. The cDNA was stored in -20°C freezers and then used for the tick species identification.

### Tick species identification

The dorsal and ventral sides of each tick were photographed with a Dino-Lite Long 90× (AM4013TL) USB-microscope (AnMo Electronics Corp., Taiwan). The pictures were analyzed in order to determine the stage and species of the tick. Due to the well-known difficulties in morphological species identification of immature *Hyalomma* ticks (13, 14), a molecular approach was applied to confirm the identifications based on tick morphology on 10 larval and nymphal tick specimens identified morphologically as *Hyalomma* sp. and considered to be representative for the entire sample. Available sequences of the different genes of *Hyalomma* species were compared in GenBank, and the mitochondrial 12S rDNA was used as a target gene.

For the molecular identification of the 10 selected ticks, standard PCR amplifications were carried out with 5 mL of cDNA and 12S rDNA primers (T1B122S and T2A12S) (15).

The PCR products were cloned and subsequently sequenced at the VIB (Flemish Institute for Biotechnology) Genetic Service Facility at the University of Antwerp, using the ABI PRISM BigDye^TM^ Terminator cycle-sequencing kit and an Applied Biosystems 3,730 DNA Analyzer. Sequencing data for the 10 *Hyalomma* ticks revealed that nine were *H. rufipes* and one was *H. marginatum* (16).

### WNV screening

A total of 729 ticks were analyzed for the presence of WNV-RNA using a one-step RT qPCR on an ABI 7,900 instrument. Eighteen of the 747 collected ticks were not analyzed due to technical problems. The binding sites of the primers were identified according to Linke et al. (17). These sites were then verified by comparing them with all available WNV sequences in GenBank. A few sequences were eliminated from the alignment due to their geographical origin and considered irrelevant in the present analysis. Degenerate primers were then designed with respect to all remaining sequences (see Table 1). Two separate nucleotide probes were used, one according to a previously described protocol (17), although redesigned as a RGB probe, to detect WNV lineage I and II, as well as a second probe that was designed to detect WNV lineage III, the so-called Rabensburg virus (see Table 1) (18).

**Table 1:** Primers and probes for WNV-specific qPCR.

WNV primer fwd.	YCT GYG TGA GCT GAC AAA CTT AGT
WNV primer rev.	GCG TTT TWG CAT ATT GAC RGC C
WNV probe 1 + 2	6-FAM-CCT GGT TTC TTA GAC ATC-MGB
WNV probe rab	6-FAM-ATC AAC AAT TAA TAC AGT GTG AGC-MGB
	Y = C/T, W = A/T, R = G/A.

A qScript one-step fast MGB RT-qPCR (Quanta, Rox) kit was used to amplify WNV RNA. Reactions were carried out with a total volume of 20 µL, containing 1.8 µL of each primer (10 µM), 0.2 µL of each probe (5 µM), and 5 µL of template RNA. Extracted RNA from a lineage 1-stem (access no. AF375045) was used as a positive control, and sterile water as a negative control. After an RT-step at 48°C for 5 min and an activation–denaturation step at 95°C for 30 sec, 45 cycles of 95°C for 3 sec and of 60°C for 30 sec were carried out.

## Results

A total of 14,824 springtime migratory birds of 78 different species were captured in mist nets at Capri bird observatory and at Antikythira bird observatory in Italy and Greece, respectively (see Table 2). From these birds, we collected 747 ticks (see Table 3). One or more ticks were found on 2.7% of the birds, and the majority of the collected ticks were larvae and nymphs of the *Hyalomma marginatum* species complex (see Tables 3 and 4). DNA sequencing of the 10 selected *Hyalomma* ticks was in accordance with the morphological identification (see the ‘Materials and Methods’ section) (16). In total, 29% of the identified ticks were larvae and 70% were nymphs (i.e. 99% of the ticks were immature).

**Table 2:** Number of birds captured and number of ticks collected from the birds.

	2009		2010		Total	
	
Locality	Birds	Ticks	Birds	Ticks	Birds	Ticks
Capri	4,924	251	4,022	158	8,946	409
Antikythira	2,529	135	3,349	203	5,878	338
Total	7,453	386	7,371	361	14,824	747

**Table 3:** Number of ticks (genus, species, and stage).

Tick genus and species	Number of ticks	Larvae	Nymphs	Adults	Stage unidentifiable
*Hyalomma marginatum* sensu lato	659	195	462	0	2
*Ixodes* spp.	28	7	17	4**	0
*Amblyomma* sp.	2	0	2	0	0
*Haemaphysalis* sp.	4	0	4	0	0
Genus unidentifiable	10	2	6	0	2
Total	703*	204	491	4	4

* 44 ticks could not be identified to genus, species, or stage due to missing photos or unidentifiable condition of the specimens.

** Ixodes frontalis.

**Table 4:** Bird species infested with ticks.

Scientific name	Common name	Number of birds	Number of ticks	Number of (%) birds infested	Mean infestation rate (Number of ticks/number of infested birds)
*Acrocephalus arundinaceus*	Great reed warbler	113	11	3 (2.7)	3.7
*Acrocephalus schoenobaenus*	Sedge warbler	452	39	13 (2.9)	3.0
*Acrocephalus scirpaceus*	European reed warbler	23	6	2 (8.7)	3.0
*Anthus trivialis*	Tree pipit	409	16	12 (2.9)	1.3
*Caprimulgus europaeus*	European nightjar	17	2	1 (5.9)	2.0
*Carduelis chloris*	European greenfinch	11	4	1 (9.1)	4.0
*Carduelis spinus*	Eurasian siskin	3	1	1 (33)	1.0
*Erithacus rubecula*	European robin	133	18	10 (7.5)	1.8
*Ficedula albicollis*	Collared flycatcher	160	6	3 (1.9)	2.0
*Ficedula hypoleuca*	Pied flycatcher	2,013	95	63 (3.1)	1.5
*Hippolais icterina*	Icterine warbler	476	7	6 (1.3)	1.2
*Hippolais pallida*	Eastern olivaceous warbler	46	2	2 (4.3)	1.0
*Lanius senator*	Woodchat shrike	144	51	18 (13)	2.8
*Luscinia megarhynchos*	Nightingale	320	35	13 (4.1)	2.7
*Motacilla flava*	Yellow wagtail	10	11	2 (20)	5.5
*Muscicapa striata*	Spotted flycatcher	1,039	7	7 (0.7)	1.0
*Oenanthe oenanthe*	Wheatear	53	21	8 (15)	2.6
*Oriolus oriolus*	Eurasian golden oriole	295	19	13 (4.4)	1.5
*Phoenicurus phoenicurus*	Common redstart	383	55	31 (8.1)	1.8
*Phylloscopus orientalis*	Eastern Bonelli's warbler	26	7	4 (15)	1.8
*Phylloscopus sibilatrix*	Wood warbler	1,239	33	30 (2.4)	1.1
*Phylloscopus trochilus*	Willow warbler	738	4	4 (0.5)	1.0
*Saxicola rubetra*	Whinchat	1,476	141	74 (5.0)	1.9
*Sylvia borin*	Garden warbler	2,191	13	11 (0.5)	1.2
*Sylvia communis*	Common whitethroat	1,245	122	68 (5.5)	1.8
*Turdus philomelos*	Song thrush	22	18	3 (14)	6.0
*Upupa epops*	Hoopoe	10	2	2 (20)	1.0
Other species		1,777	1*	1 (0.1)	
Total		14,824	747	406 (2.7)	1.8

* One tick was found on an unidentified bird species.

All positive controls from the RNA extractions, spiked with TBEV vaccine and *Borrelia*, amplified successfully when analyzed (19).

WNV RNA was not detected in any of the PCR reactions performed on the 729 tick samples. However, all negative and positive controls showed adequate curves.

## Discussion

WNV infection is considered an emerging infection, and its spread from endemic areas is facilitated by migrating birds (3, 7, 20). It has been shown that long-distance migrating passeriform birds captured in France had a 7% prevalence of WNV-neutralizing antibodies (21). However, a limited time of viremia and an impaired physical condition of WNV-infected birds would presumably reduce their potential to disperse the virus (8, 22, 23). Consequently, as viremic birds may not constitute the only method of viral dispersal from endemic to non-endemic areas, we considered the possibility that hematophagous arthropods infesting migratory birds might contribute to this process. Ticks in WNV endemic areas may attach to migratory birds. Such ticks, possibly acting as reservoirs for WNV and perhaps other pathogens, could then be carried by their avian hosts from WNV-endemic areas in Africa to Europe (8). We have recently shown that this mode of dispersal could play an important role in the spread of another tick-borne pathogen, the Crimean-Congo hemorrhagic fever virus (24).

The birds were caught during rapid northward migration on the small islands where birds normally stop over briefly just after crossing the Sahara Desert and the Mediterranean Sea. Also, most of the collected ticks were either half-fed or fully engorged nymphs, which usually attach to the host already as larvae. This indicates that most or all of these ticks had attached prior to their hosts’ migration (i.e. probably in sub-Saharan and/or North Africa). We therefore speculated that springtime migrating birds, caught at stopover localities in the Mediterranean area, may carry ticks infected with WNV from endemic areas in Africa, and thereafter transfer this potential human pathogen to regions of Southern Europe where outbreaks have recently occurred.

The results from this study do not support the above hypothesis, as none of the analyzed ticks were PCR positive for WNV. Importantly, however, epidemics of WNV have been reported recently from locations close to Antikythira, Greece, and Capri, Italy. In 2008–2009, about 20 people contracted West Nile neuroinvasive disease (WNND) in and around the Veneto region of northeastern Italy, and in 2010 the epidemic had spread to other regions of Italy, namely Sicily and Molise, both of which are close to the island of Capri (25, 26). In 2010, an outbreak of 81 cases of WNND was reported in central Macedonia in northwestern Greece (27). Thus, the two locations chosen for the collection of possible arthropod vectors for WNV are highly relevant since they are situated on birds’ migration routes between Africa and WNV epidemic areas in Europe.

Most of the collected ticks were larvae and nymphs that appeared to belong to *H. rufipes* in the *H*. *marginatum* complex. These results are similar to those of Hoogstraal et al. (28) since, in both investigations, nearly all ticks were identified as immatures that appeared to be *H. rufipes* (28). The tick infestation rate of 2.7% that was recorded in this study is also similar to that of Hoogstraal et al. (28). They found a tick infestation rate of 3.0% on birds captured in Egypt during their spring migration from East Africa to Europe.

Moskvitina et al. (29) detected WNV RNA and WNV antigen in *Ixodes pavlovskyi* and *Ixodes persulcatus*. The WNV-positive ticks had been collected from small mammals, lizards, and birds in the Tomsk Region, Russian Siberia (29).

Laboratory experiments have revealed that *H. marginatum* became infected with WNV after a blood meal from viremic hosts. The infection rates of the larval, nymphal, and adult ticks were 3, 33 and 75%, respectively. Both transstadial infection and the capacity of nymphal and adult ticks to transmit the virus to previously uninfected hosts were demonstrated (30). In Israel, ticks were collected from wild and domesticated birds and their nests, and analyzed for the presence of WNV. A total of 1.6% of *Argas arboreus* pools were positive, but none of the *Hyalomma* ticks. This is in agreement with our study. The authors suggested that some tick species may play a role in maintaining the infection in Israel (9). Reisen and coworkers (31) investigated the ability of transstadially infected *Ixodes pacificus* to transmit WNV to song sparrows and western fence lizards (31). Based on their results and previous studies, these scientists concluded that there are indications that ixodid ticks are not able to experimentally transmit WNV and therefore most likely would not be important vectors in WNV transmission cycles.

Our results do not support the hypothesis that *Hyalomma* ticks play a major role as a WNV reservoir on their avian hosts’ northward flight from Africa to Europe. The information so far obtained regarding the potential role of ticks as reservoirs and vectors is inconclusive. Further laboratory experiments on the reservoir and vector competency of different tick species are needed. Also, investigations based on a larger number of ticks of different species and geographic origins are needed to better understand the potential role of ticks in the ecology of the WNV.
